# Diversification rates indicate an early role of adaptive radiations at the origin of modern echinoid fauna

**DOI:** 10.1371/journal.pone.0194575

**Published:** 2018-03-22

**Authors:** Simon Boivin, Thomas Saucède, Rémi Laffont, Emilie Steimetz, Pascal Neige

**Affiliations:** 1 Department of Earth Sciences, University of Geneva, Geneva, Switzerland; 2 Biogéosciences, UMR 6282, CNRS, Université Bourgogne Franche-Comté, Dijon, France; Naturhistoriska riksmuseet, SWEDEN

## Abstract

Evolutionary radiations are fascinating phenomena corresponding to a dramatic diversification of taxa and a burst of cladogenesis over short periods of time. Most evolutionary radiations have long been regarded as adaptive but this has seldom been demonstrated with large-scale comparative datasets including fossil data. Originating in the Early Jurassic, irregular echinoids are emblematic of the spectacular diversification of mobile marine faunas during the Mesozoic Marine Revolution. They diversified as they colonized various habitats, and now constitute the main component of echinoid fauna in modern seas. The evolutionary radiation of irregular echinoids has long been considered as adaptive but this hypothesis has never been tested. In the present work we analyze the evolution of echinoid species richness and morphological disparity over 37 million years based on an extensive fossil dataset. Our results demonstrate that morphological and functional diversifications in certain clades of irregular echinoids were exceptionally high compared to other clades and that they were associated with the evolution of new modes of life and so can be defined as adaptive radiations. The role played by ecological opportunities in the diversification of these clades was critical, with the evolution of the infaunal mode of life promoting the adaptive radiation of irregular echinoids.

## Introduction

The uneven composition of biodiversity on Earth is evidenced by the analyses of its genetic, phenotypic, or taxonomic components at various spatial and time scales [1]. At a global scale, the 2 million extant species of organisms currently described on the planet are dominated in number by terrestrial insects and plants [1, 2]. In contrast, crustaceans and mollusks dominate taxonomic diversity of macro-organisms in modern seas, although estimating the total number of marine species remains problematic for various reasons [2, 3]. Uneven biodiversity patterns do not pertain to modern seas only. The fossil record provides evidence that marine biodiversity has always been dominated by certain clades through the Phanerozoic, but these dominant clades have not always been the same [4]. For instance, brachiopods and jaw-less vertebrates are far less diverse today than in the past, while gymnolaemate bryozoans and teleost fish have never been so speciose as they are today [3, 5]. Analyzing the dynamics of deep time macro-evolutionary changes can shed light on the origin and diversification modes of present-day biodiversity. This cannot be fully addressed by neontological studies alone because entire clades have gone extinct (including dominant components of marine communities such as trilobites and ammonites) and need to be studied from their fossil remains [6, 7, 8].

The taxonomic diversity of metazoans was greatly impacted by the five major biological crises of the Phanerozoic, which eradicated many clades and promoted the diversification of survivors during the subsequent periods of recovery [2, 9]. These major mass extinction events have received much attention from paleobiologists who, for the last decades, have dedicated their research efforts to understanding the implications of the “big five” crises for the evolution of life [10, 11, 12]. Evolutionary radiations are the counterparts of mass extinctions: they correspond to a dramatic increase in species richness and a burst of cladogeneses over short periods of time [13, 14, 15]. Compared to mass extinctions, evolutionary radiations have been little studied by paleontologists (however, see [16, 17, 18, 19]), while they have fascinated biologists for decades [14, 15, 20, 21, 22, 23, 24]. During certain intervals of the Phanerozoic, high rates of evolutionary radiation significantly modified the composition and the ecological structure of biodiversity [10]. The first and most emblematic of these was the “Cambrian explosion” that led to the emergence of the main metazoan anatomical plans [25, 26, 27]. The subsequent Great Ordovician Biodiversification Event corresponded to the diversification of the clades that dominated the world’s oceans through the Paleozoic [28, 29, 30]. During the mid-Mesozoic, the Mesozoic Marine Revolution (MMR) was at the origin of major ecological changes in the structure of benthic marine communities that still prevail in modern seas [3, 31, 32, 33]. It was marked by a significant turnover among dominant clades, the diversification of durophagous predators and predatory mollusks, the associated evolution of antipredatory morphologies, the disappearance of stalked crinoids and brachiopods from shallow marine habitats, and the increase in infaunal bioturbation activities [33, 34, 35].

Evolutionary radiations reflect multifarious and complex phenomena triggered by various biotic and abiotic factors. Although traditionally viewed as exclusively adaptive, non-adaptive radiations are now considered common phenomena in which allopatric speciation and exaptation are the main driving processes [13, 15, 17, 36, 37]. For instance, geographic radiations, in which diversification is driven by allopatric speciation due to repeated episodes of physical isolation [13, 15], have been regarded as the main generators of species flocks in the Southern Ocean [38] and are also associated with adaptive processes as in the diversification of African cichlids and Galapagos finches [39, 40]. Most studies, however, have long concentrated on adaptive radiations for explaining rapid diversification [13, 15, 41, 42, 43, 44, 45, 46] such as occurred after mass extinction events [10, 11, 12]. Adaptive radiations are evolutionary radiations in which the diversification of phenotypes is driven by adaptation and underpinned by ecological opportunities and key innovations [47, 48, 49]. They are involved in mechanisms that are prone to give access to new ecological niches and lead to evolutionary success associated with limited pressure from competition. Such mechanisms include (1) the evolution of new adaptations (key innovations) providing a marked ecological advantage over potential competitors, (2) the colonization of new environments, and/or (3) the survival of extinction events, with the last two mechanisms triggering the diversification of phenotypes due to the ecological opportunities promoted by the access to new niche spaces. Adaptive radiations lead to the combined increase in both taxonomic diversity and morphological disparity, a consequence of the diversification of phenotypes in relation with the evolution of new ecological niches [13]. Such evolutionary patterns imply an early burst in the rates of lineage diversification and phenotypic evolution, which can only be identified in deep time when studies integrate both fossil and phylogenetic data [14].

Evolutionary radiations have long been defined as adaptive when ecological diversification and adaptation were not documented [15]. Several authors have stressed the necessity to demonstrate the adaptive nature of radiations as a scientific hypothesis to be tested based on refutable grounds (see [13, 24, 50, 51]). Several criteria were stated as obligatory requirements to be fulfilled for a radiation to be defined as adaptive; they help formulate the adaptive hypothesis to be tested and possibly rejected when criteria are not satisfied. The criteria imply that candidate radiations concern monophyletic groupings, that the combined increase in taxonomic diversity and in morphological disparity must be exceptional compared to other clades, and that morphological disparity must be associated with ecological diversification because the identified innovations generate functional advantages. This last point is the most critical because demonstrating adaptation is never straightforward, especially in fossil taxa for which morphofunctional interpretations are sometimes questionable (see [18]). It also implies that phenotypic evolution must be compared with ecological diversity to determine whether the diversity of phenotypes corresponds to different functional traits and ecological features [13, 24, 50]. In deep time studies, this is facilitated when taxa have direct, extant representatives, in which the ecological advantage provided by a functional trait can be studied, and the link between the diversity of phenotypes and the diversity of ecological niches can be established [52]. This assumes that the present adaptive value is the same as earlier in the clade’s history. As claimed by Losos & Miles [24] this point remains to be explored and is obviously difficult to test [18].

Applied and theoretical studies stressed the need to explore the fossil record to uncover the deep-time macro-evolutionary processes associated with the origin and the diversification of clades [14, 18, 21, 53]. In such studies, comparison of temporal patterns of taxonomic diversity and morphological disparity of fossil clades has been shown to provide valuable insights into macro-evolutionary processes [26, 54, 55, 56, 57, 58]. The Jurassic was a time of rapid diversification for all echinoids, which differentiated into the main clades that still structure echinoid diversity and disparity in modern seas [58, 59, 60, 61]. Most of these clades have extant representatives that have been the subject of morphofunctional studies for several decades [62, 63, 64]. Originating in the early Jurassic, irregular echinoids are emblematic of the diversification of Mesozoic mobile marine faunas in soft bottom environments [60, 65, 66]. They can illustrate how key innovations associated with feeding strategies appeared during the initial diversification of marine clades during the MMR [67, 68, 69, 70]. Irregular echinoids are distinguished from “regular” forms by the general shape of the test, which evolved from circular and inflated to elongated and flat following a plan of bilateral symmetry. This evolution was associated with an increase in number and decrease in size of the spines employed for locomotion, with a decrease in size and change in shape of the peristome, with movement of the peristome forward while the periproct moved backward, and with the specialization of ambulacra [60]. All these morphological innovations are thought to have improved the efficiency of irregular echinoids in moving and feeding on the organic content of soft sediments [60, 63]. With more than 3,400 species of both fossil and extant echinoids, the clade has diversified into various marine habitats at all depths and displays high ecological diversity. Due to the anatomical innovations associated with the diversification of the clade, the radiation of irregular echinoids has long been considered adaptive [60, 61, 65, 66] but this has never been quantified or tested specifically although seminal studies have highlighted the relevance of quantifying morphological disparity and taxonomic diversity to examine rates and modes of echinoid evolution [58].

**Fig 1:**
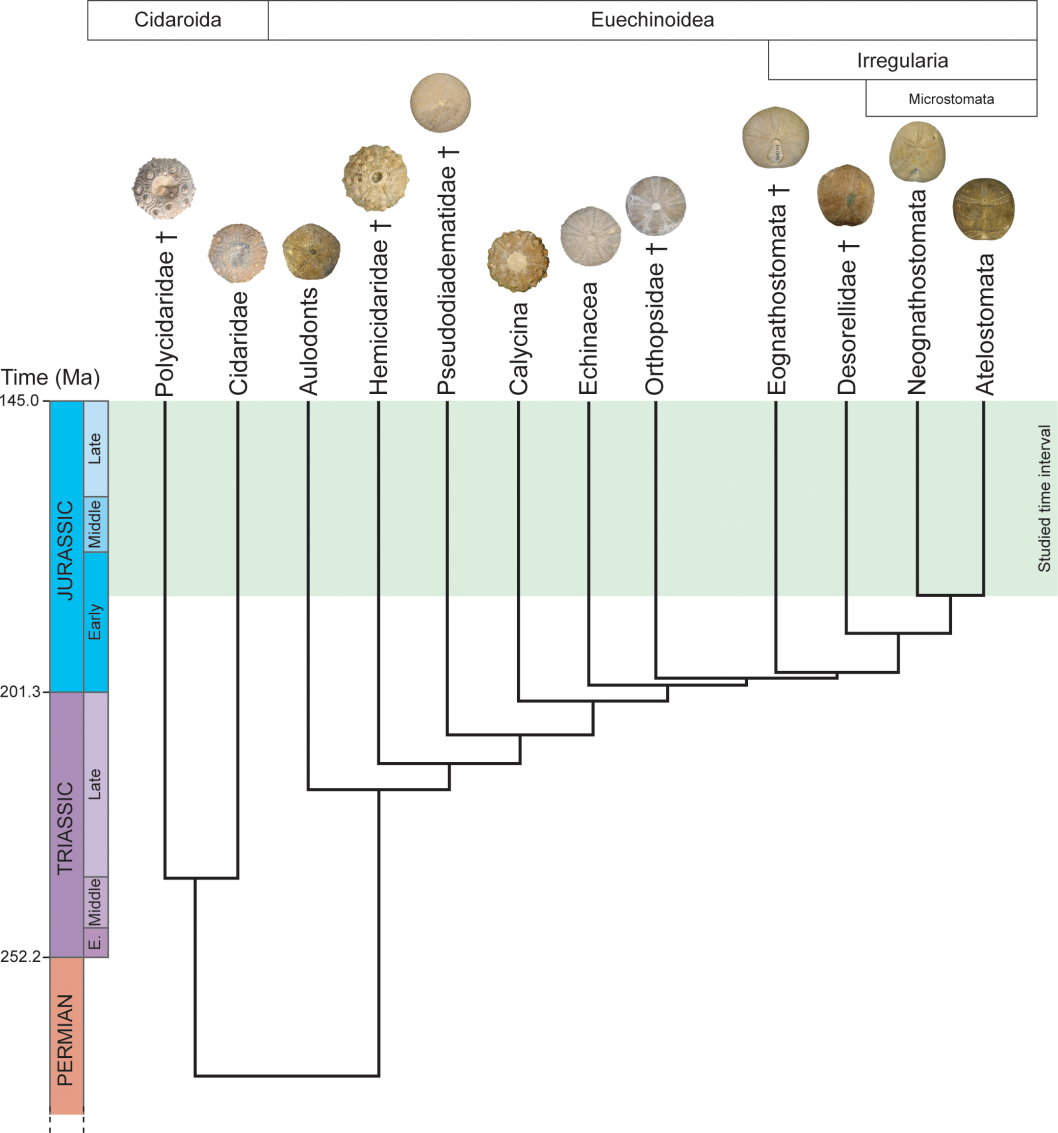
Phylogeny of main Jurassic echinoid clades. Phylogenetic relationships between clades and estimated times of divergence (in millions of years) are based on Kroh and Smith [73]. Clades are a mix of higher taxonomic ranks. Crosses indicate extinct clades. The green area corresponds to the studied time interval. Ages in millions of years Before Present (after Gradstein et al. [74]).

Based on robust morphological and molecular phylogenies previously reconstructed for the entire class Echinoidea [61, 71, 72, 73] (Fig 1), we tested the adaptive radiation hypothesis during the initial diversification of irregular echinoids. In the framework established by Losos and Miles [24] and adapted by Neige and coworkers [18] for fossils, species richness and morphological disparity were both analyzed together over a period of 37 Myrs (from 182.7 Ma to 145 Ma; Jurassic: Toarcian to Tithonian stages) when the main clades of irregular echinoids diversified. Only a few works have analyzed in echinoids the evolutionary patterns of taxonomic diversity and morphological disparity together, analyzing the disparity of the entire echinoid test at genus level using a landmark-based approach [58], or at family level based on discrete characters [70]. These significant works produced promising results and showed the relevance of such approaches to deciphering the evolutionary radiation of echinoids. Here, we focus on anatomical traits associated with feeding strategies (form and position of the peristome) and modes of life (shape of the test) and quantified disparity using a landmark- and semi-landmark-based approach at species level. We test at a fine taxonomic scale whether taxonomic diversity and morphological disparity associated with changes in feeding strategies and modes of life significantly increased in irregular echinoids compared to other clades and whether they support the adaptive radiation hypothesis.

## Materials and methods

A list of 664 fossil species of echinoids recorded worldwide (S1 File) was established based on the literature [74, 75, 76, 77]. Taxonomic relevance was optimized by discarding poorly defined or ill-known taxa as well as para-taxa established on the basis of test fragments and isolated spines [78]. Of the 910 nominal species recorded, 246 were discarded in this way. Based on the literature, the stratigraphic range of each species was documented at stage level within the Toarcian–Tithonian interval for which enough data were available for statistical inferences. In total, species belong to 12 different clades that are all monophyletic for the Jurassic based on the robust morphological and molecular phylogenies (Fig 1) previously reconstructed [61, 71, 72, 73]. Species richness values were calculated on the basis of this revised database. To assess a confidence interval for the species richness values, we performed a bootstrap procedure. For each time interval, 10,000 bootstrap samples were generated by randomly picking with replacement *n_i* species (*n_i* being the total number of species present at the *i*_th interval), and the species richness for each clade was then expressed as a ratio by dividing the number of sampled species for each clade by *n_i*. Then, the bootstrapped 95% confidence interval generated for the ratios was converted back into number of species by multiplying them by *n_i*.

The analyzed life traits are related to feeding strategy (omnivorous, algivorous, carnivorous, scavenging, deposit feeding, coarse- or fine-grained sediment feeding) and to the mode of life of echinoids relative to the sea bottom (epifaunal, semi-endofaunal, and endofaunal). The main feeding strategies of taxa could be inferred from the morphology of the peristome and the associated organs: the Aristotle’s lantern, the size, shape and position of the peristome, and the differentiation of buccal pores, interpretations relying on our knowledge of extant representatives with similar morphologies [60, 61, 64, 79, 80]. The mode of life was partly inferred based on morpho-functional arguments such as the general shape of the test and the number and size of spines employed for locomotion. It was also deduced from paleo-environmental and geological data of deposits in which fossils were reported, such as grain size of sediments from which species used to feed [61, 72].

For each species, drawings and pictures of the oral side of reference specimens available in the literature (S1 File) were used to draw the outline of the peristome, the ambitus of the test, and the position of perradial suture lines of each ambulacrum. These anatomical traits are related to echinoid feeding strategy and mode of life, and the position of the five perradial suture lines of ambulacra constitute homologous landmarks for comparison among echinoid morphologies [60]. The quality of old pictures was not detailed enough for the morphological analysis to be performed with the result that only 386 species could be drawn and analyzed. All drawings were scanned in binary bitmap mode at 300 dpi resolution.

**Fig 2:**
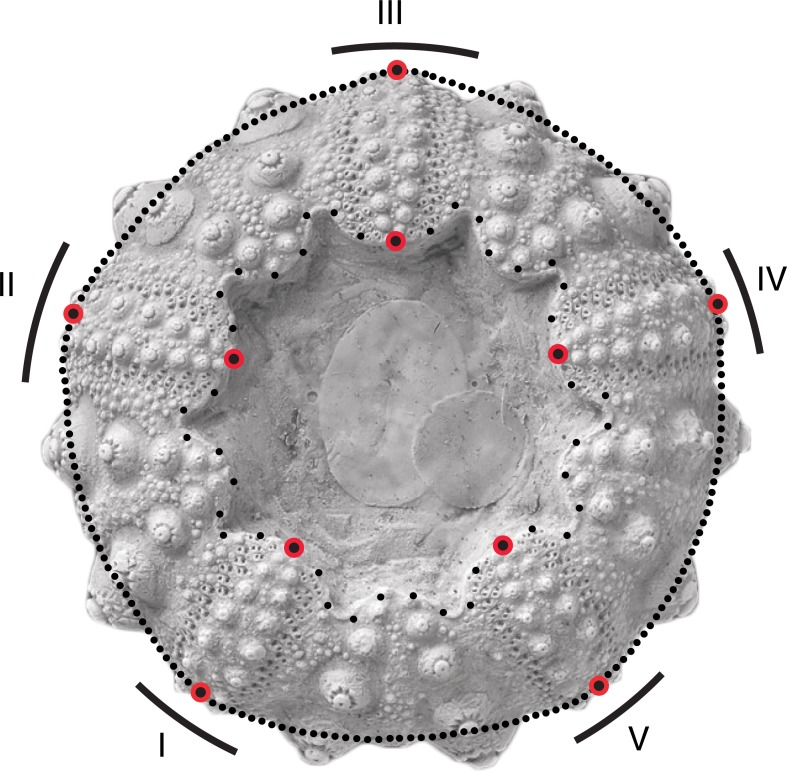
Landmark position. Landmarks (red dots) are positioned at the junction between the perradial suture lines (central line of each ambulacrum) and the peristome edge (five inner landmarks) and between the perradial suture lines and the ambitus (five outer landmarks). Roman numerals refer to the echinoid’s five ambulacra.

Drawings of echinoid outlines were depicted by a set of landmarks and semi-landmarks (Fig 2). The position of the five perradial suture lines of ambulacra being considered homologous among echinoids [60], 10 homologous landmarks of type 1 [81] were defined for analysis of the peristome outline: five at the junction between the perradial suture line and the ambitus, and five at the junction between the perradial suture line and the peristome edge. The test outline was depicted using five sets of 39 semi-landmarks [82] positioned in between the five successive landmarks. In total, the test outline was described by 195 semi-landmarks (39 × 5) and 5 landmarks (S2 File). A similar protocol was used for depicting the peristome outline with five sets of seven semi-landmarks and a total of 35 semi-landmarks and 5 landmarks (S2 File). Landmark and semi-landmark configurations were fitted using the generalized Procrustes algorithm minimizing the bending energy, and projected onto the tangent space to the mean shape [83]. Coordinates of shapes were used to calculate a Principal Component Analysis (PCA) based on the variance/covariance matrix and compute a morphospace plot using Geomorph R package version 3.0.0 [84, 85].

The first eight principal components (PCs) accounting for 95% of the total variance of the PCA were used to define morphospace and describe shape variation. Disparity was computed using two metrics: the Partial Disparity Analysis (PDA) and the Mean Pairwise Distance (MPD). PDA [54, 86] gives an overview of the relative contribution of each clade to the total disparity and its evolution through time [18]. PDA simply expresses the disparity of a given clade for a given time interval as a part of the total variance (*i*.*e*. total disparity for the considered time interval) attributable to the clade. MPD [87] is the mean of all pair-wise Euclidean distances between species of a clade computed for each time interval. Both disparity metrics were computed for clades with at least five species per time interval using the Morphospace-Disparity Analysis (MDA) package [88]. A bootstrapping procedure (*n* = 500) was performed to estimate a confidence interval associated with each value.

To test for the significance of MPD differences between clades, a set of null models was computed using Matlab®. Null distribution models were computed for each clade and each time interval following the procedure described by Losos and Miles [24] and adapted to fossil data by Neige et al. [18]. For each clade and time interval, bootstrapping was performed with 10,000 replicates by sampling with replacement m species from a total set of species. For each time interval, the total number of species corresponds to the number of species truly recorded at that time, value-centered to remove the inter-clade component of the total disparity, and m corresponds to the number of species for a given clade and time interval. Finally, MPD values of each clade and each time interval were tested against the null distributions. The test is not significant if the clade has fewer than five measured species at the time interval tested, in which case the significance of MPD is not tested. An MPD value was considered significantly large when within the 2.5% upper tail of the null distribution, and conversely, significantly low when within the 2.5% lower tail of the distribution. Values between the two thresholds were considered normal.

## Results

**Fig 3:**
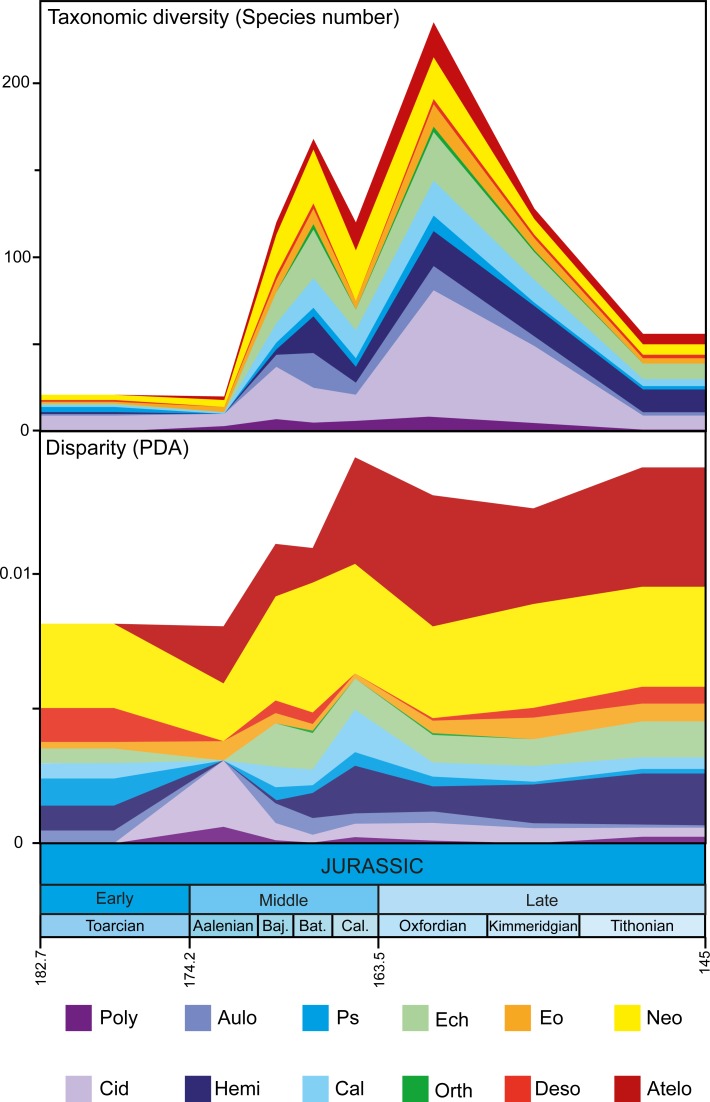
Taxonomic diversity and disparity of Jurassic echinoids. Individual contribution of echinoid clades to total taxonomic diversity and disparity levels for the studied time period. Colored areas show the values for irregular echinoid clades. Areas in white and grey are for regular echinoid clades. Poly: Polycidaridae, Cid: Cidaridae, Aulo: Aulodonts, Hemi: Hemicidaridae, Ps: Pseudodiadematidae, Cal: Calycina, Ech: Echinacea, Orth: Orthopsidae, Eo: Eognathostomata, Deso: Desorellidae, Neo: Neognathostomata, Atelo: Atelostomata.

Overall species richness of echinoids changed noticeably during the different stages of the Jurassic. Species numbers varied from just 21 species in the Toarcian (182.7–174.2 Ma) to 235 species in the Oxfordian (163.5–157.3 Ma). This overall increase in species richness was not constant during the Jurassic; it was marked by two successive surges. With few species in the ages first studied (182.7–170.3 Ma) echinoid taxonomic diversity rose sharply to a first peak in the Middle Jurassic (170.3–166.1 Ma), then it dipped before climbing again to reach its highest peak at the start of the Upper Jurassic (163.5–157.3 Ma). Thereafter, richness declined until the end of the Upper Jurassic (157.3–145.0 Ma) with just 56 species reported at the end of the period (Fig 3).

Changes in species richness differed markedly among clades (Fig 3). In the Bajocian, the diversification of the family Cidaridae pre-dated the mean peak of species richness (168 species) in the Bathonian, before it was followed by other clades in the next stages. In the Oxfordian, most clades contributed to the second peak of species richness but the Cidaridae remained the main suppliers of new species (72 species of 235 species in total). Interestingly, in two clades of irregular echinoids, the Neognathostomata and the Atelostomata, species richness did not vary in concert with this general pattern. Among the Neognathostomata, species richness increased in the Bathonian but richness values remained constant in the next stages. In the Atelostomata, species richness increased gradually until the Oxfordian with a slight dip between the Bajocian and the Bathonian. Species richness of all clades decreased at the end of the Upper Jurassic including for the Neognathostomata and the Atelostomata.

**Fig 4:**
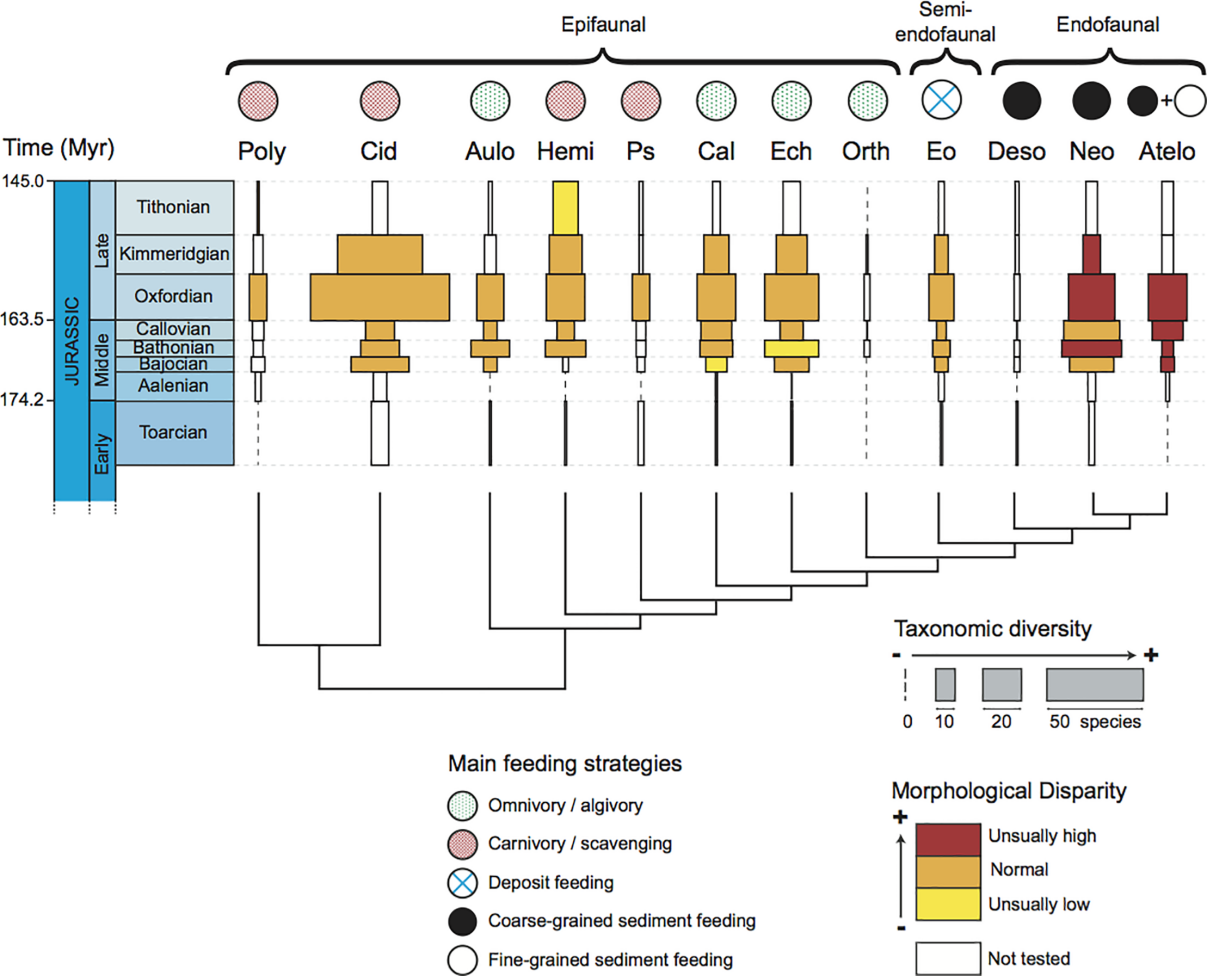
Morphospace plot of echinoid disparity. Black symbols correspond to the specimens of regular echinoids, yellow, blue, green, and red symbols represent irregular echinoids. Outlines shown for representative specimens among Cidaridae, Eognathostomata, Desorellidae, Neognathostomata, and Atelostomata. Poly: Polycidaridae, Cid: Cidaridae, Aulo: Aulodonts, Hemi: Hemicidaridae, Ps: Pseudodiadematidae, Cal: Calycina, Ech: Echinacea, Orth: Orthopsidae, Eo: Eognathostomata, Deso: Desorellidae, Neo: Neognathostomata, Atelo: Atelostomata.

Morphological disparity is represented in Fig 4 along the first two components of the PCA that account for 79.45% of the total variance. Regular echinoids are all densely clustered together in a small area of the right-hand portion of the morphospace plot. This suggests that in these clades shape evolution was conservative for the studied characters. In contrast, irregular echinoids occupy a large area of the left-hand part of the morphospace plot. The two clusters formed by regular and irregular echinoids partly overlap: this is the case for four clades: the Aulodonta and the Cidaridae, two regular echinoid clades, and the two most ancestral irregular clades, the Eognathostomata and the Desorellidae. These two irregular clades conserved common features of regular echinoids, a circular test outline and the Aristotle’s lantern that was still present in the Eognathostomata. Overall, in the smear formed by irregular echinoids, the four clades barely overlap, each clade being dispersed over a separate part of the morphospace plot. The irregular echinoids are dispersed along a morphological trend extending from the center of the morphospace plot toward the negative scores of the two PCs. This corresponds to shapes varying from circular tests with large peristomes centered in the middle of the oral side to elongated tests with small peristomes shifted toward the front of the test. Dispersion of all echinoids shows a clear arch effect. This can be interpreted as a nonlinear distribution of species along a morphological gradient due to a correlation between test shape, peristome size, position, and outline: echinoids with circular tests tend to have large circular peristomes centered in the middle of the oral side, while echinoids with elongated tests tend to have small excentric peristomes.

All regular and irregular echinoids are dispersed into separate parts of the morphospace plot during the entire period under study (Fig 4, S1 Fig). In the first stage of echinoid diversification, in the Bathonian, irregular echinoids dispersed into a new part of the morphospace while regular echinoids mostly remained in the same zone (S2 Fig). The second stage of intense diversification, in the Oxfordian, corresponds to an increase in species number in regular clades and in the Cidaridae in particular. This differs remarkably from the pattern observed in irregular echinoids, in which the total species number barely increases. There is, however, a sizeable turnover among species of irregular echinoids, with new species showing extreme morphologies and colonizing new parts of the morphospace. In the Upper Jurassic, the decline in echinoid species richness seems to be linked to random extinction with the number of shapes diminishing in all parts of morphospace while the overall surface covered by echinoids remains constant.

**Fig 5:**
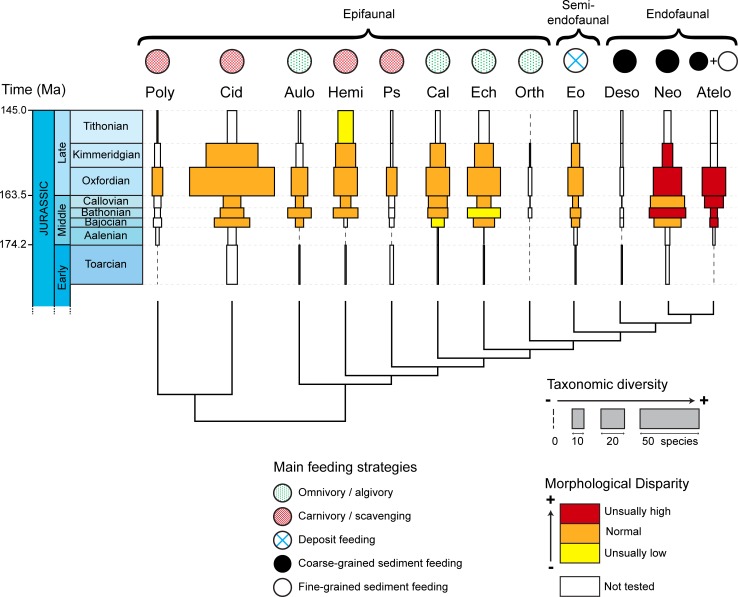
Richness and disparity levels of main Jurassic echinoid clades in relation with their feeding strategies and modes of life. The spindle diagram shows taxonomic diversity and disparity levels for the 12 Jurassic echinoid clades. For each clade, taxonomic diversity is expressed as species richness for each geological stage, the wider the rectangles, the higher the number of species. Colors correspond to the tested disparity values relative to other clades: unusually high (red), normal (orange), unusually low (yellow), not tested due to lack of data (white). Main feeding strategies and modes of life were interpreted for each clade. Ages in million of years Before Present (after Gradstein et al. [73]).

Disparity values are congruent with the general pattern of morphospace occupation described above (Fig 3, S3 Fig). Different metrics to quantify the disparity were computed using the MDA package. However, because they all displayed the same patterns (S3 Fig) we focused on MPD and PDA. During the studied stages, irregular echinoids were the main contributors to total echinoid disparity (PDA). During the Toarcian, irregular echinoids already contributed to over 50% of the PDA and the ratio increased to 68% by the end of the Jurassic. When comparing disparity values among clades, two irregular clades clearly stand apart from the other ones with relatively high disparity values: the Neognathostomata and the Atelostomata. In the other two irregular clades, the Desorellidae were highly disparate during the Bajocian and the Bathonian alike but disparity values could not be computed for the other stages due to gaps in the fossil record. Finally, the Eognathostomata had average disparity levels compared to regular echinoids. Overall, disparity remained constant during the studied period except in one irregular clade: the Atelostomata, in which disparity increased from the Aalenian to the Oxfordian. To check for the significance of disparity differences between clades, we compared the measured disparity values with a null model of disparity distribution (S4 Fig). Only two clades of irregular echinoids had unusually high disparity values (higher than expected by chance): the Neognathostomata during the Bathonian, Oxfordian, and Kimmeridgian, and the Atelostomata from the Bajocian to the Oxfordian (Fig 5). In contrast, three regular echinoid clades showed unexpectedly low disparity values: the Hemicidaridae during the Tithonian, the Calycina during the Bajocian, and the Echinacea during the Bathonian.

**Fig 6:**
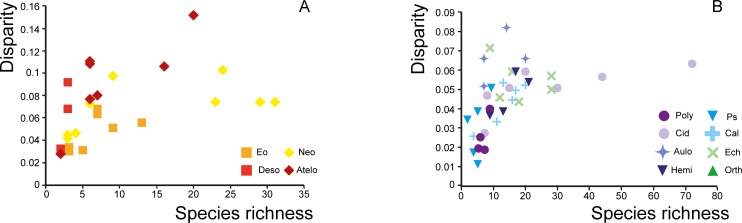
Biplot of species richness and disparity. Species richness (expressed in number of species) is plotted against disparity (expressed as the Mean Pairwise Distance) for each clade and each time interval. The four clades of irregular echinoids are plotted separately from the others (respectively A and B).

At the scale of the entire class, there is no clear relationship between clade mean diversity and disparity values (S5 Fig). In regular echinoids, almost all clades exhibit similar disparity values while taxonomic diversity values differ remarkably. Conversely, most irregular echinoids have high disparity levels, which are independent of their respective taxonomic diversity values. In contrast, within most clades, taxonomic diversity and disparity values tend to covary during the Jurassic, with high disparity values corresponding to high diversity values (Fig 6, S3 Fig). This is the most marked in Atelostomata, in which covariation between taxonomic diversity and disparity values is significant (R^2^ = 0.6137, *p* = 0.037) (Fig 6, S3 Fig). Diversity and disparity clearly increased from the Aalenian to the Oxfordian, except for a slight dip during the Bathonian. Then both diversity and disparity decreased in concert until the end of the Jurassic. In the Neognathostomata, taxonomic diversity and disparity covary but the highest peaks were shifted in time. Taxonomic diversity and disparity initially increased together from the Aalenian to the Bathonian when diversity reached its peak. Then diversity steadily declined until the end of the Jurassic while disparity increased, peaking in the Oxfordian and remaining high until the end of the period. Covariation between diversity and disparity also occurred in the Cidaridae (Fig 6), but the increase in disparity remained moderate while diversity values were very high in the Upper Jurassic (S3 Fig).

## Discussion

Three echinoid clades display taxonomic diversity values that are congruent with a pattern of evolutionary radiation, that is, a sudden, relative increase in species number compared to other clades: one clade of regular echinoids, the Cidaridae, and two clades of irregular echinoids, the Neognathostomata and the Atelostomata. In the Cidaridae, species richness increased in the Oxfordian but it was accompanied by a moderate increase in disparity, at least with respect to the morphological characters analyzed (Fig 6, S3 Fig). The association of a burst of taxonomic diversity with a moderate increase in morphological disparity suggests a pattern of non-adaptive radiation, which has rarely been documented in the literature [13, 89]. In contrast, in the Atelostomata, the fossil record provides evidence of a simultaneous increase in both diversity and disparity from the Bathonian to the Oxfordian. This combined increase in taxonomic diversity and morphological disparity is consistent with patterns of adaptive radiation because morphological disparity is linked here to characters that are inferred to be ecologically functional [18, 90]. In the third clade, the Neognathostomata, the evolutionary pattern seems more complex. Initially, diversity and disparity increased in concert (Aalenian and Bathonian) and then diversity decreased until the end of the Jurassic (Kimmeridgian), whereas disparity remained unusually high (Figs 3 and 5, S2 Fig). These diverging evolutionary trends between disparity and diversity in the second part of the time interval could be congruent with a subsequent stage of specialization. After an initial interval of radiation that was putatively underpinned by adaptive processes (see below), the clade subsequently evolved toward extreme morphologies represented by taxa with elongated test shapes and an anteriorly displaced peristome. This phenomenon was accompanied by the selective extinction of “intermediate” forms represented by ancestral taxa (members of the so-called “galeropygids”), which were common in the lower and Middle Jurassic and displayed circular test shapes with the peristome centered in the middle of the oral side [79, 91]. This is congruent with recent results obtained at the family level by Hopkins and Smith [70] who showed the selective extinction of the majority of intermediate forms linking regular and irregular echinoids as well as those linking Atelostomata and Neognathostomata.

Patterns of diversity and disparity evolution in the irregular clades Neognathostoma and Atelostomata are typical of adaptive radiations: combined increases in both species richness and morphological disparity. The high disparity level early in the history of Atelostomata has already been shown by Eble [58]. More recently, Hopkins and Smith [70] showed that peaks in taxonomic diversity were associated with major shifts in morphological characters associated with feeding strategies in the two clades. In the Neognathostoma, an adaptive radiation can be suspected at the origin of the clade only, while in the Atelostoata it can be assumed over a longer period of time, from the Bathonian to the Oxfordian (Fig 5). At this point, if the adaptive radiation hypothesis cannot be rejected, the functional significance of morphological evolution must be analyzed with respect to the environments colonized to check whether ecological opportunities could have been associated with the evolution of new phenotypes [13]. Adaptive radiations are initiated when new traits originate [21] and are associated with the evolution of taxa for new diets and habitats (i.e. ecology) [92]. They may be either associated with the colonization of new habitats or with a gain in efficiency in using the resources of the newly colonized habitats, or both [13, 21]. Interestingly, species of Neognathostomata and Atelostomata have been interpreted as being mostly endofaunal and sediment swallowers [66, 79] based on the appearance of morphological innovations present in extant taxa and on geological evidence documenting the paleoenvironments that fossil specimens inhabited [60, 66, 72]. These modes of life and feeding strategies as well as the associated morphological characters were unknown in echinoids before the Jurassic and evolved in three clades only: the Neognathostomata, the Atelostomata, and the Desorellidae, a clade of irregular echinoids that is restricted to the Jurassic and is too poorly-known for its disparity to be analyzed (Fig 5, S4 Fig). The congruence in variation patterns between taxonomic diversity and morphological disparity that are typically associated with adaptive radiations, the appearance of a new mode of life, and the evolution of a new feeding strategy associated with new morphologies, suggest that key innovations and new ecological opportunities triggered the diversification of the clade in soft bottom environments. During the Middle Jurassic, ecological opportunities were also promoted by the expansion of carbonate platforms and the significant increase in carbonate sediment volumes that provided endofaunal species with potential new habitats and new niche space [66, 93, 94].

Morphological innovations associated with the radiation of the two clades concern the peristome and associated organs: the loss of the Aristotle’s lantern, the decrease in size of the peristome, the change in shape and position of the peristome, and the appearance of buccal pores corresponding to the evolution of new tube feet [64]. These morphological innovations corresponded to the possibility for the echinoids to improve efficiency in the ingestion of the organic matter present in organic-poor sediments, either in between the sediment particles or as a coating around the grains [60, 63]. The evolution of the characters implies that the echinoids acquired the ability to ingest large quantities of sediment into their digestive tract to digest the organic content, or pick up the detritus present in between the sediment grains, initially in coarse-grained (Neognathostomata and oldest Atelostomata) and then in fine-grained (other Atelostomata) sediments (Fig 5). Associated with the evolution of the feeding structures, the general shape of the test evolved from circular and inflated to elongate and flat to facilitate the locomotion of echinoids on and in soft sediments and improve the efficiency in covering large areas of organic-poor sediments. This was associated with the increase in number and decrease in size of the spines employed for locomotion. Considering the morphological characters tested and their functional significance, the hypothesis of an adaptive radiation cannot be rejected to explain the origin of the Neognathostomata and Atelostomata, which gave rise to the majority of present-day echinoids.

Originating in the Toarcian and in the Aalenian respectively, both clades diversified mainly in the Bajocian. The shift in time between the origination and the diversification of the clades does not imply that there is no causal relationship between the evolution of the key innovation and the radiation. Radiations are not necessarily concomitant with the acquisition of key innovations but can be more or less delayed depending on processes associated with the modification of the key traits [19], or depending on niche availability. Interestingly, the Atelostomata have been the only echinoids to adapt to feeding upon fine-grained sediments. This was made possible by the functional evolution of the echinoid tube feet that evolved the capacity to pick up organic particles from fine-grained sediments [60, 63, 64]. Atelostomata then further evolved the capacity to ingest huge quantities of sediments of different grain sizes and digest the organic content, reflecting that key innovation and new ecological opportunities triggered the diversification of new species over a longer interval of time.

Many studies have documented the temporal patterns of taxonomic and morphologic changes that occurred in various groups of marine organisms during the MMR [31, 33, 34, 35]. They stressed the importance of biotic factors, among which escalation as a trigger of taxonomic and ecological diversification [34, 35, 68]. Several case studies have been extensively analyzed, including the diversification of major groups of durophagous predators that evolved the ability to crush shells and the evolutionary response of mollusks that developed antipredatory morphologies while other taxa migrated to the deep sea to avoid the growing predation pressure [33, 34]. More recently, macro-evolutionary turnovers have been documented among ammonite clades in the early and mid-Mesozoic, along with the diversification of Osteichthyes at the expense of Chondrichthyes [12, 18]. The present study illustrates the role played by ecological opportunities for the diversification of clades and the evolution of the infaunal mode of life promoting the adaptive radiation of certain irregular echinoids during the MMR. The diversification of irregular echinoids was not associated with the decline of “regular” clades because new ecological opportunities and the newly colonized habitats prevented large-scale niche saturation. Therefore, the diversification of irregular echinoids led to a general increase in both the taxonomic diversity and the morphological disparity of the entire class during the Mesozoic [60, 65]. This result is not consistent with other case studies. Benson et al. [90], for instance, suggested that high evolutionary rates lasted over long periods of time during the initial diversification of avian-dinosaurs and birds, whereas other (non-avian dinosaurs) lineages showed declining evolutionary rates after early-burst diversification events in the Jurassic. On the contrary, not all of the echinoid clades that did not radiate during the MMR subsequently went extinct, and some of them even experienced later diversification events at the end of the Mesozoic and in the Cenozoic [65]. These contrasting results call for more studies dissecting diversification patterns in deep time to analyze the macro-evolutionary patterns and processes associated with major diversification events.

## Supporting information

S1 File Dataset of echinoid occurrence records. Occurrence records of the 664 fossil species of echinoids recorded worldwide for the eight geological stages of the studied time period based on the literature [72, 73, 74, 75]. Type species names are in bold. First Appearance Datum (FAD) and Last Appearance Datum (LAD) given in million of years Before Present (after Gradstein et al. [71]). References are the source data used for morphometric analyses.
(XLSX)

S2 File Dataset of landmark and semi-landmark coordinates. Coordinates of landmarks (LM) and semi-landmarks (SLM) used to depict ambitus and peristome outlines of each species.
(XLSX)

S1 Fig Morphological space detailed for each echinoid clade. All specimens of echinoid are plotted in light grey along the first two components of the PCA (see Fig 4). The respective specimens of each clade are highlighted in dark (regular clades) or in color (irregular clades).
(EPS)

S2 Fig Morphological space of echinoid disparity plotted for each geological stage. For each stage, only species occurring at that time period are plotted in the PCA.
(EPS)

S3 Fig Echinoid disparity and diversity detailed for each clade and each geological stage. Grey shaded bars of histograms show diversity values expressed as species richness for each geological stage (left axis, in number of species). Red curves show disparity values expressed as Mean Pairwise Distance for each geological stage (right axis). Error bars correspond to 95% confidence intervals computed by bootstrapping (500 replicates). Ages in million of years Before Present (after Gradstein et al. [71]).
(EPS)

S4 Fig Testing for the significance of disparity differences. To test for the significance of differences in disparity between clades null distribution models were computed for each clade and each time interval following the method developed by Losos and Miles [21] and Neige et al. [18]. The measured disparity values expressed as Mean Pairewise Distances (MPD) were compared to the range of null distribution values (grey bars) to test whether they can be considered unusually high (grey circles) or low (open circles). No test was performed for clades with less than five species per time period. Poly: Polycidaridae, Cid: Cidaridae, Aulo: Aulodonts, Hemi: Hemicidaridae, Ps: Pseudodiadematidae, Cal: Calycina, Ech: Echinacea, Orth: Orthopsidae, Eo: Eognathostomata, Deso: Desorellidae, Neo: Neognathostomata, Atelo: Atelostomata.
(EPS)

S5 Fig Compared total diversity and disparity values for each clade. Plot of morphological disparity expressed as the Mean Pairwise Distance (MPD) against taxonomic diversity in number of species for the studied time interval. Poly: Polycidaridae, Cid: Cidaridae, Aulo: Aulodonts, Hemi: Hemicidaridae, Ps: Pseudodiadematidae, Cal: Calycina, Ech: Echinacea, Orth: Orthopsidae, Eo: Eognathostomata, Deso: Desorellidae, Neo: Neognathostomata, Atelo: Atelostomata.
(EPS)
