# Correction: Diversification rates indicate an early role of adaptive radiations at the origin of modern echinoid fauna

**DOI:** 10.1371/journal.pone.0196375

**Published:** 2018-04-19

**Authors:** Simon Boivin, Thomas Saucède, Rémi Laffont, Emilie Steimetz, Pascal Neige

Fig 4 is incorrect. The authors have provided a corrected version here.

**Fig 4:**
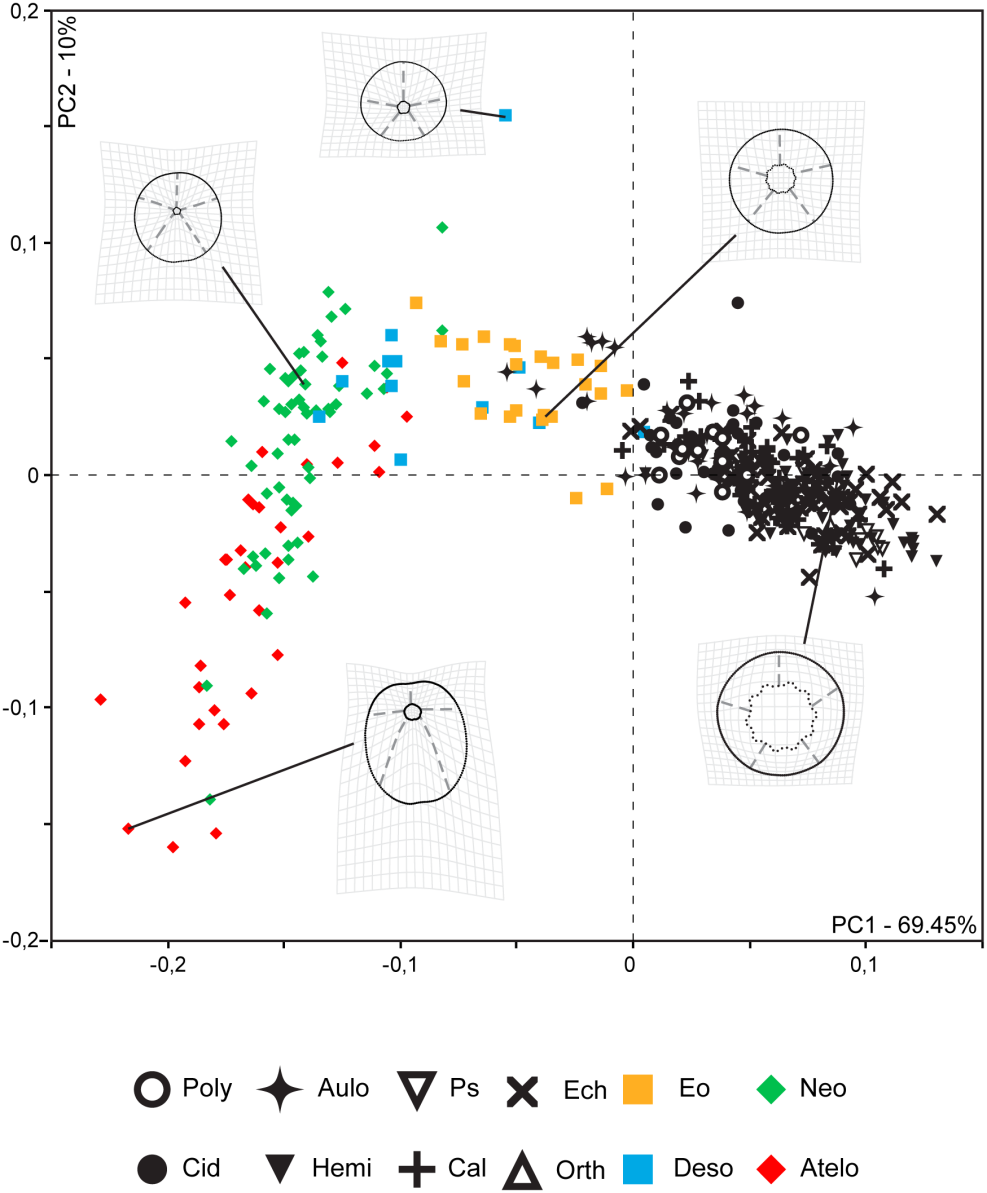
Morphospace plot of echinoid disparity. Black symbols correspond to the specimens of regular echinoids, yellow, blue, green, and red symbols represent irregular echinoids. Outlines shown for representative specimens among Cidaridae, Eognathostomata, Desorellidae, Neognathostomata, and Atelostomata. Poly: Polycidaridae, Cid: Cidaridae, Aulo: Aulodonts, Hemi: Hemicidaridae, Ps: Pseudodiadematidae, Cal: Calycina, Ech: Echinacea, Orth: Orthopsidae, Eo: Eognathostomata, Deso: Desorellidae, Neo: Neognathostomata, Atelo: Atelostomata.
